# Intra-articular Hip Injection Using Anatomical and Radiological Landmarks Without the Use of Ultrasound or Radiological Guidance

**DOI:** 10.7759/cureus.23581

**Published:** 2022-03-28

**Authors:** Christos Yiannakopoulos, Nikolaos Sachinis, Jennifer Oluku, Spilios Dellis

**Affiliations:** 1 Orthopedic, IASO Hospital, Athens, GRC; 2 Physical Education and Sports Science, National and Kapodistrian University of Athens, Athens, GRC; 3 Orthopedics and Trauma, Georgios Papanikolaou General Hospital, Thessaloniki, GRC; 4 Radiology, Guy's and St Thomas' National Health Service (NHS) Foundation Trust, London, GBR

**Keywords:** guided injections, anatomical landmark, hip arthritis, hip radiograph, hip aspiration, hip injection

## Abstract

Introduction: Intra-articular hip injections are routinely performed under sonographic or fluoroscopic guidance in order to improve accuracy. The purpose of this study was to evaluate the safety and accuracy of a hip injection technique that does not require the use of fluoroscopic or ultrasound guidance and can be performed in the clinic. A combination of radiographic and anatomic landmarks was used in order to perform the hip injection, based on the use of simple hip radiographs.

Methods: In this prospective study 35 patients with hip osteoarthritis or femoroacetabular impingement were included. All patients underwent intra-articular hip joint injection using the technique we describe. The injection location was determined based on measurements performed on hip radiographs using as reference points fixed anatomical landmarks, i.e., the anterior superior iliac spine (ASIS), the cephalic, and caudal femoral head-neck junctions. The vertical distance between the ASIS and the greater trochanter and the horizontal distance between the two head-neck junctions, and the vertical line were also measured. The accuracy of the injection was assessed using ultrasound examination before and after the injection in order to verify intra-articular fluid injection.

Results: Intra-articular hip joint injections using the described non-guided technique were successful in 33 of 35 (94.3%) patients without any complications.

Conclusion: Hip injections can be performed with high accuracy without the need for radiological or ultrasound guidance using the described technique. The combination of radiological and anatomical landmarks to perform intra-articular hip injections is safe, cost-effective, and accurate.

## Introduction

Intra-articular injections in the hip joint are commonly performed for diagnostic and therapeutic purposes [1-3]. Hip joint aspiration assists in the diagnosis of intra-articular conditions, such as septic arthritis, crystalline arthropathy, and periprosthetic infection. Hip joint injections are usually performed using fluoroscopy or ultrasound guidance in order to improve their targeting accuracy with excellent results [1-3]. The use of anatomical landmarks is a feasible alternative to imaging-guided injections in the hip and several studies showed that injections can be performed safely and effectively based only on anatomical landmarks [4-7]. However, intra-articular hip injections performed solely on the basis of anatomical landmarks have lower success rates as compared to image-guided injections mainly because accurate identification of the anatomical landmarks can be challenging especially in large or obese patients [5,8].

The purpose of our study is to describe a non-imaging guided hip joint injection technique incorporating measurements from radiological and anatomical landmarks and to assess its targeting accuracy.

## Materials and methods

This prospective study included patients who underwent intra-articular hip joint injections for therapeutic purposes. All patients provided written consent for the procedure and the study was approved by the Institutional Review Board (IRB) of the hospital where these procedures were performed. All patients had one injection and there were no consecutive injections. Patients who had a previous hip operation or surgical incisions around the hip and patients who had a history of hip trauma were excluded. A total of 12 patients was excluded from the study for the above reasons. We included 35 patients who received 35 intra-articular hip injections. Twenty-seven patients (77.1%) suffered from symptomatic hip osteoarthritis, diagnosed clinically and using hip radiographs and eight patients (22.9%) suffered from a hip labrum tear due to femoroacetabular impingement, diagnosed clinically and with the use of MRI. Nineteen patients were women (mean age 47.2 years, range 28-61 years) and 16 were men (mean age 49.9 years, range 32-71 years). The average weight was 84 ± 11.8 kg and the average BMI was 28 ± 5.1 kg/m^2^. The BMI range was from 17.8 to 31.4. Three patients were having a BMI over 30. All patients received an intra-articular corticosteroid (2 mL of triamcinolone acetate 40 mg/mL) and local anesthetic (8 mL bupivacaine 0.5%) injection.

Injection technique

**Figure 1:**
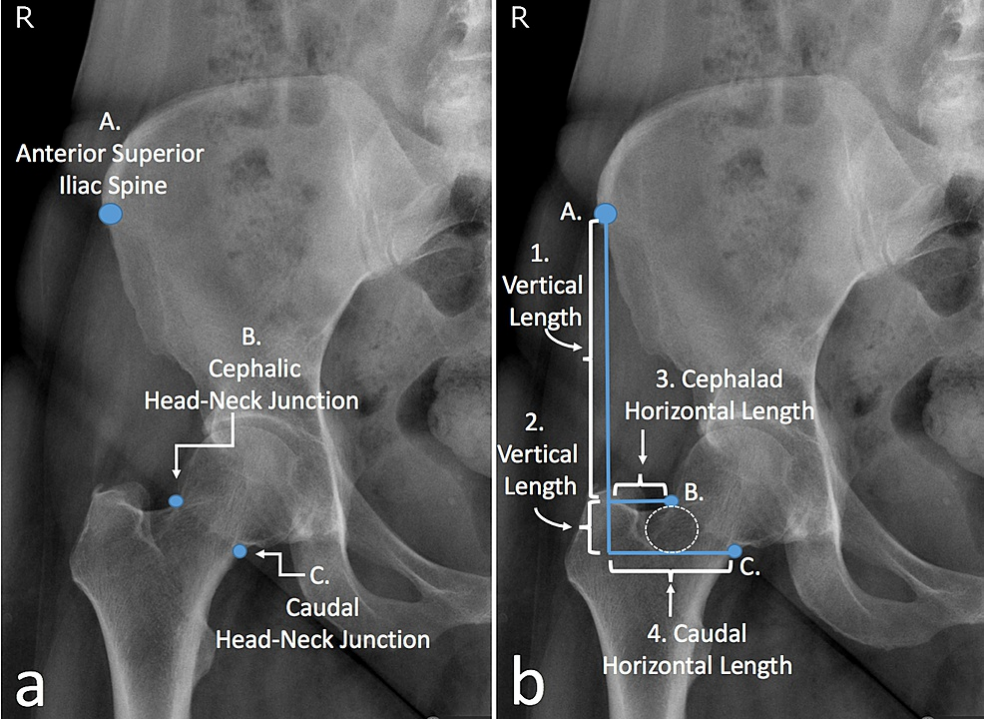
Identification of the anatomical reference points. On an anteroposterior hip radiograph with 100% magnification, three anatomical reference points are recognized: point A, the anterior superior iliac spine (ASIS), point B, the cephalic and point C, the caudal femoral head-neck junction (A). Three lines are drawn: a vertical line from the ASIS to the greater trochanter and two horizontal lines from points B and C connecting them to the vertical line. The preferred injection site is the anterolateral head-neck junction (circle, B).

Prior to the injection, three points were located on a pre-injection anteroposterior hip radiograph with 100% magnification: point A, the anterior superior iliac spine (ASIS); point B, the cephalic femoral head-neck junction; and point C, the caudal femoral head-neck junction (Figure 1a). Three lines were drawn: a vertical line originating at the ASIS (Lines 1 and 2, Figure 1b) and two horizontal lines connecting the vertical line and points B and C (Lines 3 and 4, Figure 1b). The injection site was the anterolateral head-neck junction (circle, Figure 1b), midway between the two horizontal lines, 1 cm inferior to point B.

**Figure 2:**
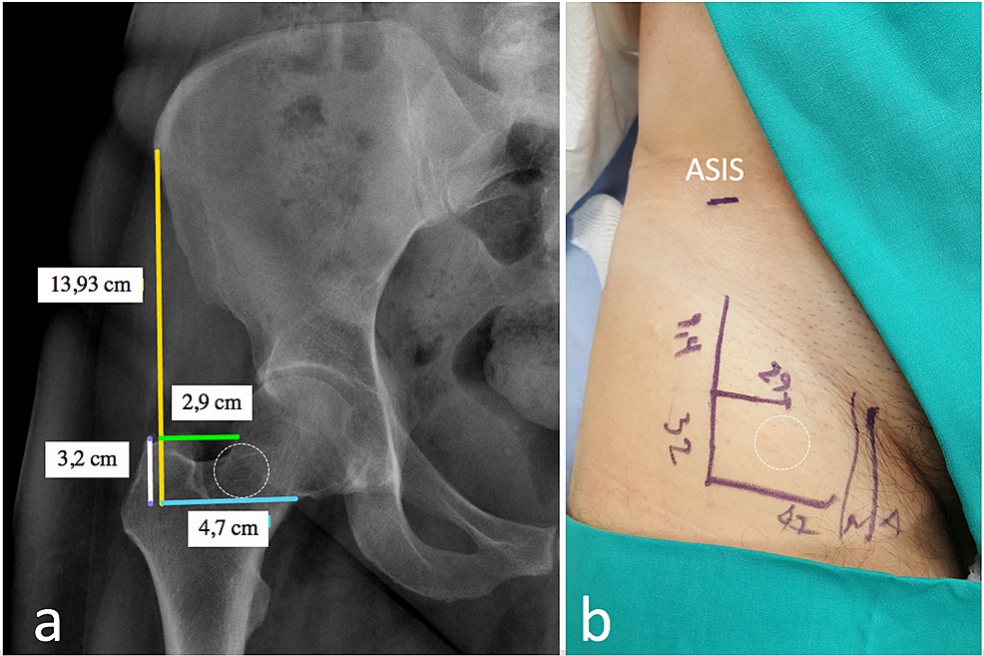
Estimation of the infection site. The respective distances from the ASIS vertical line and points B and C are measured with accuracy using commercially available software (A). Clinical photograph of an actual patient prior to the injection (B). All previously measured landmarks are drawn on the skin of the groin area.

The needle was oriented perfectly vertical. The respective distances from the ASIS line and points B and C can be measured using any DICOM viewer software (Figure 2a) and replicated on the patient based on the anatomical landmarks (Figure 2b).

**Figure 3:**
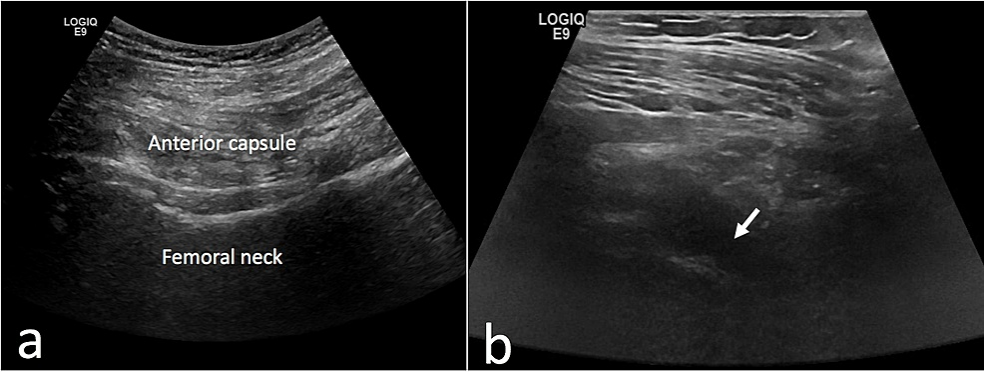
Ultrasound examination of the hip before and after the injection. Ultrasound examination of the hip with the probe at an oblique sagittal position along the femoral neck. The hyperechoic femoral head, neck, and anterior capsular recess are recognized prior to the injection with no apparent fluid collection (A). Following the intra-articular injection, there is hypoechoic fluid accumulation in the anterior femoral neck area (B).

Ultrasound examination of the hip was performed prior to the injection using a 7 MHz linear transducer before and after the injection in order to confirm the accuracy of the injection. The ultrasound probe was rotated to an oblique sagittal position and aimed at the umbilicus. The femoral head, neck, and the anterior capsular recess were recognized (Figure 3a) verifying the absence of a hip effusion. Ultrasound examination was performed after the injection. The presence of intra-articular hip joint fluid after the injection was considered an indication of successful accomplishment of the injection (arrow, Figure 3b).

The patient was placed in the supine position with the lower limb in neutral rotation, without knee flexion, and the area was prepped and draped. External rotation of the hip should be avoided because it brings the neurovascular bundle closer to the injection site [9]. The location of the ASIS and the greater trochanter (GT), as well as the position of the femoral artery below the middle of the inguinal ligament, were marked on the skin (Figure 2b).

Further details regarding injection technique are discussed below. A vertical line from the ASIS was drawn on the skin according to the pre-injection radiographic measurements. At the predetermined points, the horizontal lines to the cephalic and caudal head-neck junctions were drawn on the skin. The injection site was distal to point B (cephalic head-neck junction), in an area with a diameter of about 2 cm (circle, Figure 2b). The hip joint capsule surrounds the femoral neck and is attached anteriorly to the intertrochanteric line, superiorly to the base of the femoral neck, posteriorly 1 cm above the intertrochanteric crest, and inferiorly to the femoral neck near the lesser trochanter, so the injection point is intracapsular.

A superficial local anesthetic wheal was performed at the planned needle entry site using 2 mL lidocaine 2%. A 20 Gauge, 6 cm long spinal needle was used, angled 30º distally and medially and inserted in the injection point. The penetration of the hard hip capsule and the anterior femoral neck bone can be clearly felt. After tactile confirmation of contact with the anterior femoral neck, the stylet is removed from the needle and the injection is performed. Although it is not necessary the intra-articular position of the needle can be confirmed by injecting 10-20 ml of normal saline in the joint. Return of the plunger to its original position confirms the intra-articular position of the needle. Fluid backflow after removing the syringe also indicates that the needle is in the correct intra-articular position. All the injections were performed by the same surgeon. In order to verify the accuracy of the injection, ultrasound examination was performed again immediately after the injection to evaluate the presence of intra-articular hip joint fluid (arrow, Figure 3b). All the ultrasounds were performed by the same doctor who is an experienced musculoskeletal radiologist. The duration of the procedure was recorded. The starting point was after the standard prepping and draping just before starting to mark the bony landmarks and the ending point was the completion of injection and withdrawal of the needle. The patient’s discomfort was assessed using a numeric visual analog scale (VAS) for pain (0 to 10). 

## Results

Injections performed using a combination of radiological and anatomical landmarks were successful in 33 patients (94.28%) as verified by the post-injection ultrasound examination. In the two unsuccessful injections, the fluid was found to be located distal to the joint, along the lateral aspect of the femoral neck. In none of these patients, the fluid was at clinical proximity to neurovascular structures traversing anterior or posterior to the joint. The mean procedural time was 3.6 ± 1.7 minutes and the mean VAS pain score during the procedure, representing patient discomfort, was 2.4 ± 0.9. Immediate pain relief, within the first 10 minutes after the injection, was reported in 82.85% of the hips (29/35) and delayed relief, within the next 8 hours, in 17.15% of the hips (6/35).

All patients were followed up by the surgeon who performed the injection. The follow-up took place at two weeks, four weeks, and three months after the injection. There were no complications associated with the injection procedure, such as femoral or lateral femoral cutaneous nerve injury, hematoma formation, or infection.

## Discussion

Hip joint injections have significant diagnostic and therapeutic applications. They are used by a large number of medical professionals including orthopedic surgeons, rheumatologists, and radiologists. Various techniques have been described using ultrasound or fluoroscopic guidance [5,7].

A hip joint injection is technically difficult because the hip lies deeply with only the GT of the femur palpable. The hip joint capsule is thick with limited distensibility. Additionally, there is a risk of the femoral nerve and femoral artery injury as they are located beneath the middle part of the inguinal ligament. The bifurcation of the common femoral artery takes place in the lower third or distal to the lower border of the femoral head in 90% of patients and thus the optimum location for a hip joint injection lies at a safe distance from it. The femoral artery shows a consistent anatomical course, running over the femoral head in 92% of cases, while in 99% of cases the bifurcation of the common femoral artery is distal to the middle of the femoral head [10].

Imaging-guided, intra-articular joint injections are more accurate than landmark-guided injections independently of the anatomical site [7]. However, the need for ultrasound or radiographic guidance complicates their use as they require specialized equipment, personnel, and training. Radiation exposure in hip joint injections using fluoroscopic guidance is a rather serious disadvantage because it can be quite significant. Exposure is related to the body mass index and means fluoroscopy times were reported to be 17.4 ± 9.9, 17.5 ± 11.4, and 19.1 ± 13.4 seconds in average weight, overweight, and obese patients, respectively [11].

A number of studies have assessed the efficiency of intra-articular hip injections using anatomical landmarks, without the use of imaging. This technique while having significant advantages, has demonstrated high rates of failure. Singh et al. assessed 87 patients who had 100 hip injections through an anterior approach using anatomical landmarks and reported a failure rate of 33%. Injections performed by consultants in non-obese patients proved to have the highest success rates (87.9%) [12].

Ultrasound guidance eliminates the risk of radiation exposure but requires expensive equipment and significant training and experience. Other possible drawbacks of the ultrasound-guided technique are the suboptimal visualization of structures that are deeply located in obese individuals, the acoustic shadow of the bone, and the limited visualization when the needle is thin or is inserted at a steep angle. Hip injections using only anatomical landmarks circumvent the disadvantages of imaging-guided injections but have a high percentage of failure. Accurate location of the anatomical landmarks is not always reliable, even if performed by expert surgeons [13]. In one study comparing palpation of anatomical landmarks with ultrasound verification, only one out of 30 expert hip arthroscopists was able to palpate the ASIS and the GT, the so-called lighthouse of the hip, with an error of less than 10 mm [13]. Palpation is problematic in obese or muscular individuals and in patients with structural hip deformity or flexion deformity [4]. Needle misplacement is also likely when the greater trochanter is high-riding and in hips with increased femoral version [8].

Mei-Dan et al. [14] assessed 55 patients who had 55 hip injections also using anatomical landmarks and an anterior approach and demonstrated 93% accuracy [14]. Diracoglu et al. [15] performed 57 injections, Ziv et al. [8] 40 injections and Mauffrey et al. [6] 20 injections through the lateral approach using anatomical landmarks and reported a success rate of 50.9%, 77.5% and 95%, respectively. Kurup and Ward [4] used an anterolateral approach using landmark guidance in 43 patients and reported an accuracy rate of 65%. A systematic review and meta-analysis conducted by Hoeber et al. [16] compared ultrasound-guided and landmark-guided hip joint injections concluding that ultrasound-guided injections are significantly more accurate.

We evaluated a technique of hip joint injection using the combination of anatomical and radiological landmarks, based on plain hip radiographs. Pre-injection anteroposterior hip radiographs with 100% magnification were used, projecting the measurements on the anterior groin area to avoid palpation-based variability.

## Conclusions

We described an imaging-free technique for intra-articular hip injections that combines radiological and anatomical landmarks and is based on a simple hip radiograph. This technique can be easily used in day-to-day practice in order to perform intra-articular hip injections.

Our proposed technique only requires a hip radiograph, which is a cheap, safe, and well-established investigation commonly used in orthopedic practice. This technique is safe, effective, and cheap; does not require sophisticated equipment, and most importantly, prevents both patient and staff from radiation exposure.
